# Emergence of human avian influenza A(H7N9) virus infections in Wenshan City in Southwest China, 2017

**DOI:** 10.1186/s12879-020-4858-6

**Published:** 2020-02-19

**Authors:** Li Jiang, Xiaonan Zhao, Wen Xu, Xuehua Zhou, Chunrui Luo, Jiunan Zhou, Xiaoqing Fu, Yaoyao Chen, Duo Li

**Affiliations:** 1 The First Affiliated Hospital of Guizhou University of Traditional Chinese Medicine, Guiyang, Guizhou China; 2 Yunnan Provincial Center for Disease Control and Prevention, 158 Dongsi street, Kunming, Yunnan 650022 People’s Republic of China; 3 Wenshan Prefecture Center for Disease Control and Prevention, Wenshan, Yunnan, China

**Keywords:** Influenza a(H7N9) virus, Emerge, Human infection, Wenshan

## Abstract

**Background:**

The emergence of human infection with avian influenza A(H7N9) virus was reported in Wenshan City, southwestern China in 2017. The study describes the epidemiological and virological features of the outbreak and discusses the origin of the infection.

**Methods:**

Poultry exposure and timelines of key events for each patient were collected. Samples derived from the patients, their close contacts, and environments were tested for influenza A(H7N9) virus by real-time reverse transcription polymerase chain reaction. Genetic sequencing and phylogenetic analysis were also conducted.

**Results:**

Five patients were reported in the outbreak. An epidemiological investigation showed that all patients had been exposed at live poultry markets. The A(H7N9) isolates from these patients had low pathogenicity in avian species. Both epidemiological investigations of chicken sources and phylogenetic analysis of viral gene sequences indicated that the source of infection was from Guangxi Province, which lies 100 km to the east of Wenshan City.

**Conclusions:**

In the study, a sudden emergence of human cases of H7N9 was documented in urban area of Wenshan City. Chickens were an important carrier in the H7N9 virus spreading from Guangxi to Wenshan. Hygienic management of live poultry markets and virological screening of chickens transported across regions should be reinforced to limit the spread of H7N9 virus.

## Background

Influenza A viruses belong to the *Orthomyxoviridae* family which comprises seven genera: Influenza virus A, B, C, and D, *Thogotovirus*, *Isavirus and Quaranfilvirus* [1, 2]. The influenza A viruses are further classified into subtypes based on the antigenicity divergence and sequence comparison of the two viral surface glycoproteins, HA and NA. Currently, 18 HA and 11 NA subtypes have been identified [3, 4]. According to epidemiological features, influenza in humans can be described as seasonal, pandemic or human-avian influenza. At present, seasonal A influenza is caused by the viruses of subtypes A(H1N1) and A(H3N2). Influenza pandemics occur when new strain/subtype of virus emerges. Large population will be infected causing high levels of mortality. The disease produced by the avian influenza viruses (AIV) that transmitted across species to humans is called human-avian influenza. The two most important AIV causing human threats are the H5N1 and H7N9 subtypes. The ongoing circulation of these viruses continues to pose a pandemic threat due to their rapid geographical expansion and genetic diversity, and may eventually the adaptation to humans which may result in human-to-human transmission [5].

Since March 2013, novel strains of H7N9 AIV have emerged and spread rapidly across mainland China. As of 4December 2019 H7N9 AIV has caused six epidemic waves with 1568 laboratory-confirmed cases. However, the H7N9 virus is still confined to China, with the exception of a few cases who had history of travelling to China [6]. In the first four waves, the geographic distribution of H7N9 outbreaks was much more limited to the southeast coastal area from near the Yangtze River delta (YRD) to farther south around the Pearl River delta (PRD). Few cases in inland areas were reported but the geographical distribution of the epidemic had clearly expanded [7, 8]. The fifth epidemic of H7N9 AIV infection in China broke out on October 1, 2016, and continued to spread during 2017. As of September 30, 2017, 766 people had been virologically confirmed, accounting for nearly half of all human cases reported since 2013 [9]. In wave five, there were eight provinces with newly emerged human H7N9 AIV infection, namely, including Chongqing, Gansu, Inner Mongolia, Shaanxi, Shanxi, Sichuan, Tibet, and Yunnan Province, which are areas of Western or Northern China [10].

**Fig. 1:**
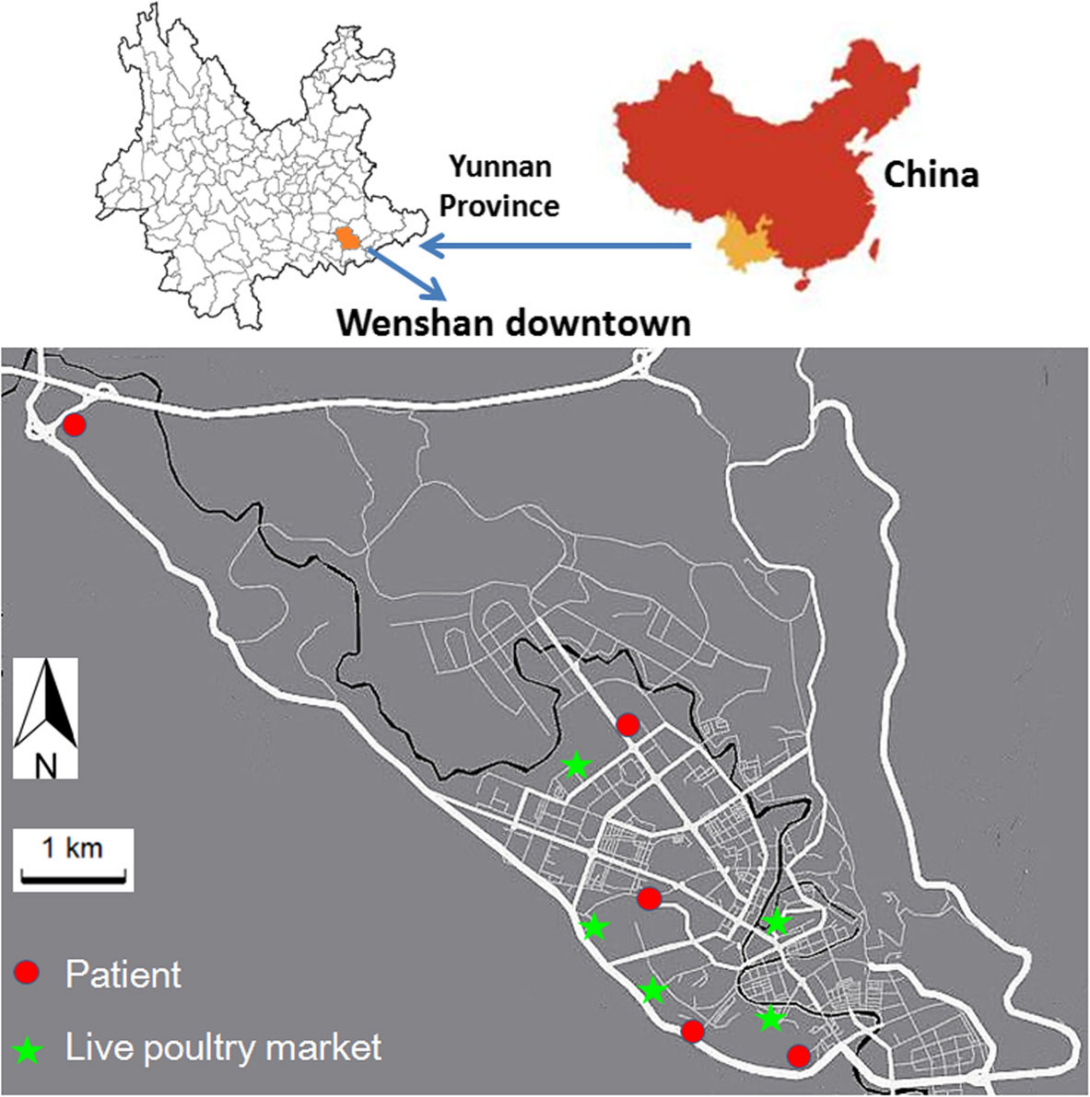
The geographic distribution of confirmed human cases of avian influenza A(H7N9) virus in Wenshan City, Yunnan Province, China in 2017. (The map layers were provided and permitted to use in the study by China Center for Disease Control and Prevention)

Yunnan Province, southwest China (Fig. 1), did not experience of human infection during the first four epidemic waves of the H7N9 virus. However, two imported human H7N9 cases were detected in Kunming City, the capital of Yunnan, in February 2017 [11]. Four months later, indigenous human cases of H7N9 virus infection were demonstrated in the urban area of Wenshan City of the province. The abrupt emergence of human infection has attracted considerable attention on the current prevention and control strategies.

In this study, we investigated the epidemiological characteristics of patients infected with H7N9 AIV, compared the genetic features of local viral isolates with other viral strains in the 5th epidemic wave to determine the origins and evolution of the H7N9 virus, and discussed the effectiveness of current control measures in Wenshan City.

## Methods

### Study area

Wenshan City is located in the southeastern Yunnan province (Fig. 1), which spans ~ 3064 km^2^ and has a population of 500,000, ~ 34.2% of whom live in urban areas. The city is approximately 200 km from Kunming City in the north and 100 km from the border with Guangxi Province in the east. It is also an important traffic node connecting Kunming and Guangxi.

### Epidemiological investigation

Information about patient exposure to poultry was obtained by interviewing the patients or the patient’s parents. The timelines of key events during hospitalization were extracted from the medical records. Throat swabs of patients were tested for influenza virus by real-time reverse transcription polymerase chain reaction (real-time RT-PCR) using the specific primers provided by the Chinese National Influenza Center [12]. The primers, reaction system and cycling steps were summarized Additional file 2: Table S1.

According to the Prevention and Control Guideline for Human Avian Influenza A(H7N9) Virus Infection [13], close contacts were identified as individuals who had provided care to, had been living with or had potentially been directly exposed to respiratory secretions or bodily fluids of the patient. Influenza-like symptoms (fever of ≥38 °C and/or coughing) were monitored daily among the close contacts for 7 days. Asymptomatic human H7N9 AIV infection has been reported from Guangzhou Province [14]. Thus, throat swabs were collected from all close contacts and tested for influenza virus by real-time RT-PCR.

Environmental samples containing poultry feces, poultry cage surface swabs and live poultry market (LPM) sewage from poultry markets in downtown Wenshan were collected and tested by real-time RT-PCR, which was the method used for patient diagnosis.

### Molecular analysis

Full genome sequences of the human H7N9 viruses from Wenshan City were determined by either China Center for Disease Control and Prevention (CDC) or Yunnan CDC, and the sequences were deposited in the GISAID database (platform.gisaid.org), with accession numbers listed in Additional file 3: Table S2. These viruses were examined for the key amino acid residue changes in each gene segment to assess their receptor specificity, drug resistance and mammalian adaptations.

### Genomic and bioinformatic analysis

Seven hundred thirty-five full HA gene sequences of H7N9 in the 5th epidemic wave (viruses collected from 1 October 2016 through 30 September 2017) were downloaded from the GISAID database. These sequences were aligned and compared using the MEGA 7 program (University of Pittsburgh, PA). Phylogenetic trees were constructed using the maximum likelihood method in the MEGA software, and 1000 replicates were used for bootstrap analysis. Only bootstrap values that were greater than 70 are shown in the results. All trees were rooted using the virus A/Shanghai/2/2013 (H7N9).

## Results

### Epidemiological investigation

**Fig. 2:**
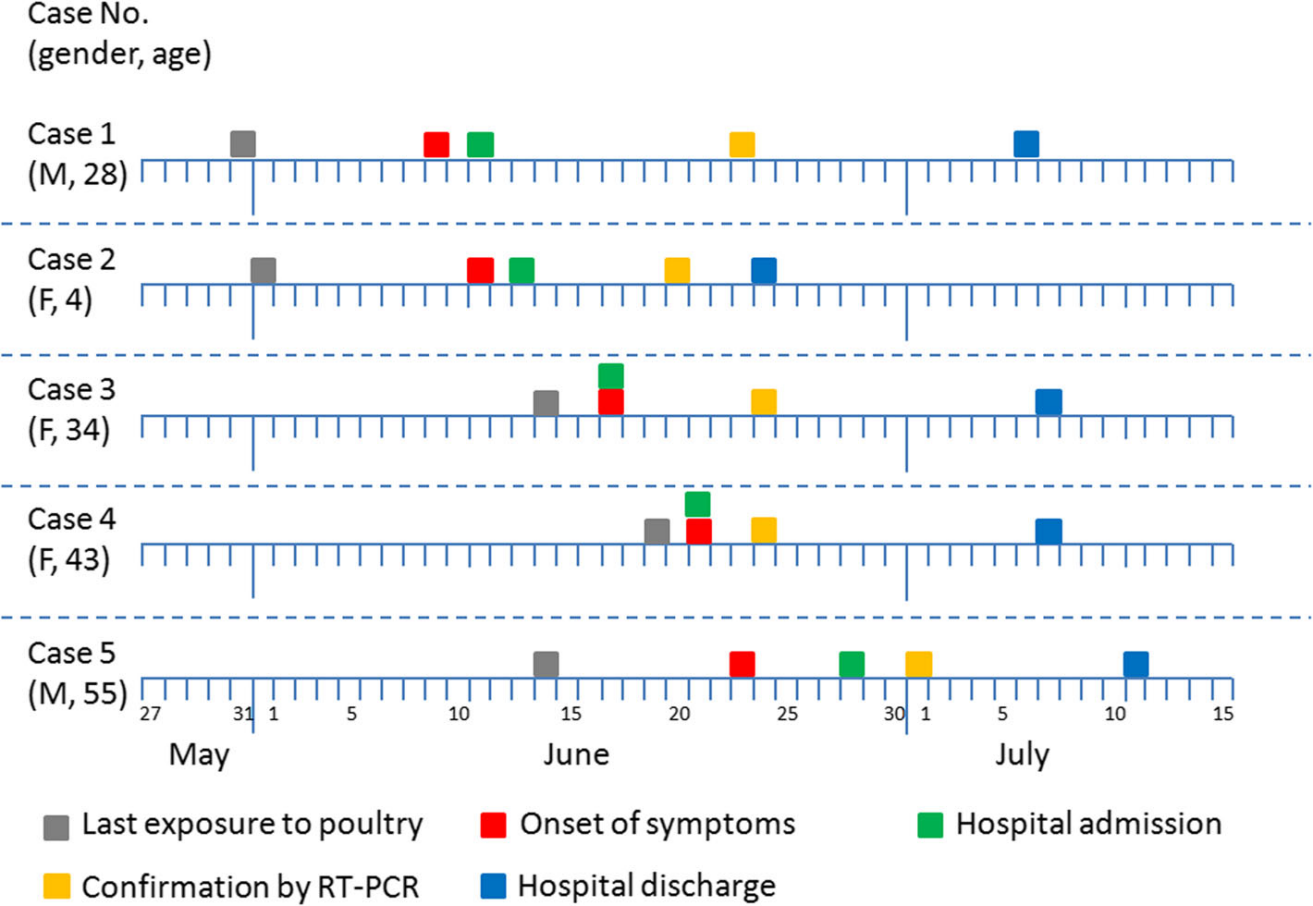
The timeline of key events for the five human cases of avian influenza A(H7N9) virus in Wenshan City, Yunnan Province, China in 2017

The index patient (Case 2) was a 4-year-old girl from downtown Wenshan. She developed high fever (39 °C) on June 11, 2017. Two days later, she was admitted to a local hospital with acute tonsillitis. The patient was diagnosed as an influenza-like illness case, and a throat swab sample was collected and sent to the influenza network laboratory of the local CDC for routine surveillance. Avian influenza H7N9 virus RNA positivity was confirmed by the local CDC on June 20 using real-time RT-PCR. The symptoms were mild and the case was discharged from the hospital on June 24. During hospitalization, she was treated with antibiotics and febrifuge drugs without antiviral treatment. The patient had visited a local LPM on June 1, 10 days before the onset of symptoms (Fig. 2).

Since the confirmation of the index patient, 4 additional human H7N9 infections were continually detected in June and July. Case 1 had high fever (40 °C) on June 9 and developed pneumonia. The case was confirmed as H7N9 virus infection via routine influenza surveillance work on June 23. Due to reports of two human H7N9 infections, the local CDC declared an H7N9 outbreak in the city. Nucleic acid detection of H7N9 virus was required by local hospital clinicians when they encountered patients with pneumonia not responding to antibiotic treatment. Cases 3, 4 and 5 were actively found by hospital doctors and confirmed by sending a swab sample to the local CDC. All the additional 4 cases had been exposed to local LPM (Figs. 1 and 2). Furthermore, an epidemiological investigation showed that the chickens sold at the city markets were mainly originally transported from the adjacent Guangxi Province.

Medical tracing was performed on 78 identified close contacts. All the close contacts were followed up for seven consecutive days, and no flu-like symptoms were noted among these people. Throat swabs were collected from the close contacts, and all tested negative for the H7 subtype.

There are five LPMs in downtown Wenshan (Fig. 1). Six hundred forty-three samples containing poultry feces, poultry cage surface swabs and market sewage samples were collected from all five of the LPMs from June 20 to July 6. Thirty-nine of the samples tested positive for the H7 subtype by real-time RT-PCR and covered all five of the markets. However, the positive chicken and environmental samples failed to yield a virus isolate. After the positive detection in the LPMs, control measures including closure and disinfection of LPMs were performed from June 21 through July 7. Two hundred five environmental samples were collected again from these markets from July 13 through 21. All tested negative for the H7 subtype by real-time RT-PCR.

### Viral amino acid substitutions

Full viral genomes were determined in four of five cases (except Case 5). These sequences were analyzed to identify the critical amino acid (aa) residue changes that may indicate their receptor specificity, drug resistance and mammalian adaptations. The HA proteins of all four isolated viruses presented a basic aa (KGR↓G) at the cleavage site, suggesting low pathogenicity in poultry. Substitutions at V186 and L226 in the HAs were identified, indicating an increased binding ability to human α 2,6-linked sialic acid receptors. No neuraminidase inhibitor (oseltamivir) resistance markers were present in the NA genes. All strains contained N31 in the M2 proteins, indicating amantadine resistance. Substitutions appeared at V368 in the PB1 protein, which was associated with an increased transmission in ferrets (Table 1).

**Table 1:** Molecular characteristics of the H7N9 viruses isolated from humans in Wenshan 2017

Gene	Function	Mutation	Case No. Virus isolates			
			1A/Yunnan/32294/2017	2A/Yunnan/wenshan01/2017	3A/Yunnan/32291/2017	4A/Yunnan/32293/2017
HA	Increased binding to human type influenza receptor	G186V	V	V	V	V
		Q226L	L	L	L	L
	Enhanced virulence in poultry		KGR↓G	KGR↓G	KGR↓G	KGR↓G
NA	Antiviral oseltamivir resistance	A246T	A	A	A	A
		R292K	R	R	R	R
PB2	Increased virulence in mice	E627K	E	E	E	E
	Enhanced transmission in guinea pigs	D701N	D	D	D	D
	Species-specific signature positions	K702R	K	K	K	K
PB1	Increased transmission in Ferret	I368V	V	V	V	V
PA	Species-specific signature positions	V100A	V	V	V	V
	Increase the polymerase activity in mice	L336M	L	L	L	L
	Species specific signature positions	K356R	R	R	R	R
		S409N	N	N	N	N
NS1	Altered virulence in mice	D92E	D	D	D	D
	altered antiviral response in host	N205S	S	S	S	S
		G210R	G	G	G	G
M	Anti-viral amantadine resistance	S31N	N	N	N	N

Notes: The HA residues are based on the H3 numbering system and the NA residues are based on the N2 numbering system. Other internal genes were numbered from the start codon

Abbreviations: *HA* hemagglutinin, *NA* neuraminidase, *NS* nonstructural protein, *PA* polymerase acidic protein, *PB*1 polymerase basic protein 1, *PB*2 polymerase basic protein 2, ↓ cleavage site

### Phylogenetic analysis

**Fig. 3:**
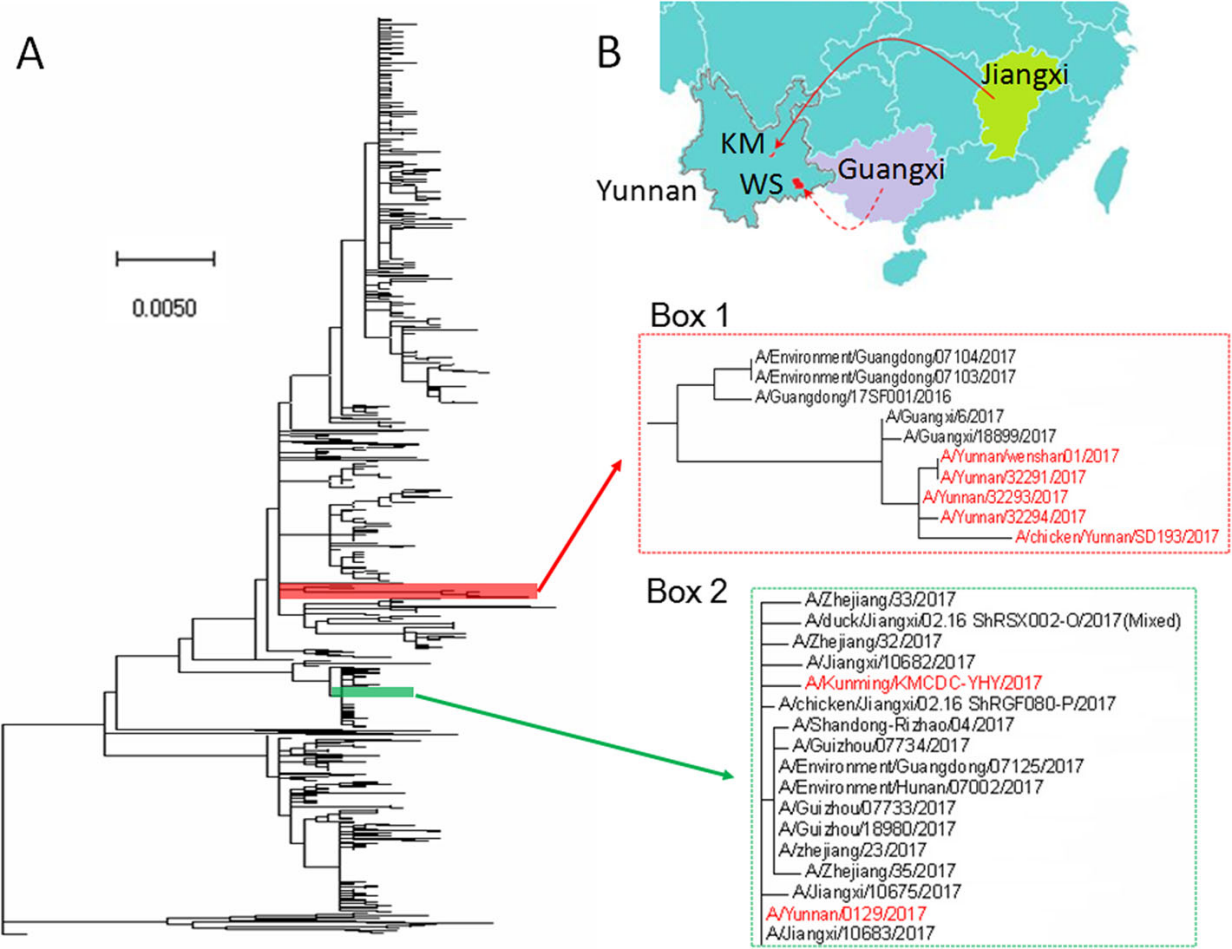
Phylogenetic tree of the HA gene of avian influenza A(H7N9) virus in the 5th epidemic wave. **a** H7N9 viruses isolated from Yunnan province are shown in detail in the dotted boxes and are colored in red. Box 1, the isolates in Wenshan City; box 2, the imported strains in Kunming City. The tree is rooted with virus A/Shanghai/2/2013. **b** A partial map of China indicating the routes of viral spread leading to outbreaks in cities of Kunming and Wenshan. Abbreviations: KM, Kunming City; WS, Wenshan City. Kunming strains imported from Jiangxi Province are shown as a solid line. Wenshan strains imported from Guangxi Province are shown as a dotted line. (The map layers were provided and permitted to use in the study by China Center for Disease Control and Prevention)

Phylogenetic analyses revealed that the HA genes of the four isolates from Wenshan patients were clustered in the same clade and were genetically similar to those from the infected patients in Guangxi Province in 2017. Additionally, the HA genes of the patient isolates were closely related to that of the isolate from the chicken sample in Wenshan City (Fig. 3 A, Box 1, Additional file 1: Figure S1). However, the HA genes of the isolates from the cities of Wenshan and Kunming belonged to different branches in the phylogenetic tree (Fig. 3 A, Box 2). The Kunming strains were imported from Jiangxi Province. In addition, the Wenshan strains were transmitted from Guangxi Province by imported chickens (Fig. 3 B).

### Identity of nucleotide sequences in viruses from Wenshan and Guanxi patients

Because the HA genes of the Wenshan viral strains were similar to those of the two isolates (A/Guangxi/6/2017 and A/Guangxi/18899/2017) from Guangxi Province by the above phylogenetic analysis, all eight viral gene segments were compared between the two isolate sources to determine the identities. The results showed that the nucleotide identity was over 99% in external genes (HA and NA) and was from 96 to 99% in six internal genes (Table 2). The results showed that these viruses share a high degree of nucleotide sequence similarity.

**Table 2:** Percent nucleotide identity of influenza A(H7N9) viruses from Wenshan and Guanxi patients

Viral segment and virus strain	Proportion (%) of sequence identity	
	A/Guangxi/6/2017	A/Guangxi/18899/2017
PB2		
A/Yunnan/wenshan01/2017	99.78	99.74
A/Yunnan/32291/2017	99.78	99.74
A/Yunnan/32293/2017	99.82	99.78
A/Yunnan/32294/2017	99.65	99.60
PB1		
A/Yunnan/wenshan01/2017	98.84	98.71
A/Yunnan/32291/2017	98.84	98.71
A/Yunnan/32293/2017	98.89	98.75
A/Yunnan/32294/2017	98.80	98.66
PA		
A/Yunnan/wenshan01/2017	99.11	99.86
A/Yunnan/32291/2017	99.11	99.86
A/Yunnan/32293/2017	99.11	99.86
A/Yunnan/32294/2017	99.11	99.86
HA		
A/Yunnan/wenshan01/2017	99.76	99.70
A/Yunnan/32291/2017	99.76	99.70
A/Yunnan/32293/2017	99.82	99.76
A/Yunnan/32294/2017	99.76	99.70
NP		
A/Yunnan/wenshan01/2017	99.60	99.60
A/Yunnan/32291/2017	99.60	99.60
A/Yunnan/32293/2017	96.41	96.41
A/Yunnan/32294/2017	96.76	96.76
NA		
A/Yunnan/wenshan01/2017	99.50	99.50
A/Yunnan/32291/2017	99.50	99.50
A/Yunnan/32293/2017	99.50	99.50
A/Yunnan/32294/2017	99.50	99.50
M		
A/Yunnan/wenshan01/2017	97.00	97.00
A/Yunnan/32291/2017	97.00	97.00
A/Yunnan/32293/2017	97.00	97.00
A/Yunnan/32294/2017	97.00	97.00
NS		
A/Yunnan/wenshan01/2017	99.76	99.76
A/Yunnan/32291/2017	99.76	99.76
A/Yunnan/32293/2017	99.64	99.64
A/Yunnan/32294/2017	99.76	99.76

## Discussion

In this investigation, we identified the emergence of human infection with H7N9 AIV in Wenshan, Southwest China and presented epidemiological characteristics of the infection, genetic evolution of the virus, and origin of the transmission.

The H7N9 outbreak in Wenshan occurred in June. Similar to what has been observed for other provinces with newly emerged H7N9 cases (except Sichuan and Chongqing), human cases have been reported since April and mainly in May and June. Live poultry or poultry products transported from previously affected provinces caused the gradual spread of H7N9 AIV. Thus, the peak of human infections in the provinces with newly emerged cases was delayed by 4 months compared to eastern China [15]. All cases in the Wenshan H7N9 outbreak had a history of LPM exposure in urban area. This is different from the H7N9 outbreaks in neighboring Guangxi Province, where showed a higher proportion of backyard poultry exposure among human cases [16]. Therefore, the Wenshan outbreak was quickly brought under control by closure of LPM and environmental disinfection.

HA genes of isolates from Wenshan mainly clustered with viruses from Guangxi provinces. However, the isolates from other newly emerged provinces clustered with different isolates from various regions of China [15]. The phenomenon reflected the H7N9 AIV was spread by multiple routes in the whole country. All the four isolates from Wenshan patients contained a single basic aa substitution (KGR↓G) at the cleavage site, which has been associated with low pathogenicity in avian species. A highly pathogenic H7N9 AIV variant emerged and caused 28 human infections in seven provinces (Guangdong, Taiwan, Guangxi, Hunan, Shaanxi, Hebei, and Henan) in the Wave Five [17]. However, there was no significant difference in fatality and severity in humans infected with either low or high pathogenicity H7N9 AIV [18]. Ativiral resistance-conferring R292K mutations on NA protein were detected from eastern China in the Wave Five [17, 19]. However, all of the four Wenshan isolates did not contain any of the amino acid substitutions that are known to confer reduced inhibition by the NA inhibitor class of antivirals. These results indicate that neuraminidase inhibitor antiviral drugs could still be effective for the treatment. Amino acid changes D627K and D701N in PB2 have been considered to be critical adaptations and virulence factors for infecting mammals. They have much higher proportions in human-isolated H7N9 viruses than in avian-isolated forms in all five waves [20]. In the Wenshan outbreak, no isolate presented K627 and N701 mutations, which reflects a direct cross-species transmission of H7N9 AIV from chickens to humans.

In this study, both epidemiological investigations of chicken sources and phylogenetic analyses of the genetic similarity of virus strains indicated that the source of infection is from Guangxi Province. Guangxi is adjacent to Guangdong Province in the east, and it is one of two H7N9 origin centers (the other one is YRD) with the highest cumulative numbers of reported incidences of human infection with H7N9 AIV since 2013 [21]. In wave five, human H7N9 infections were also first reported in the two H7N9 origin centers, YRD and PRD, followed by surrounding regions [20, 22]. The first laboratory-confirmed case in Guangxi was imported from neighboring Guangdong on January 2017. Until the last case reported on June 9, H7N9 AIV spread to ten of 14 prefecture level cities with 27 cases in Guangxi [16]. Soon after that, the H7N9 outbreak was detected in Wenshan City. The transmission route spanned a long geographical region with spread from east to west.

Chickens were an important carrier in the H7N9 virus spreading from Guangxi to Wenshan. Similar findings from Chongqing also demonstrated a high positive rate of H7N9 infection in live chickens transported from other provinces between February and June 2017 [23]. However, due to the low pathogenicity of the H7N9 virus causing asymptomatic infections and circulating silently in chickens, it was impossible to predict a possible human infection by infected chickens without any laboratory tests [24, 25] Thus, virological screening in chickens before cross-regional transportation is necessary to prevent H7N9 virus spread.

## Conclusion

Our study documented that the sudden emergence of human cases of H7N9 in Wenshan in 2017 was due to exposure to imported live poultry at LPMs, indicating that hygienic management of LPMs and virological screening of chickens transported across regions should be reinforced to limit the spread of H7N9 virus.

## Supplementary information

**Additional file 1: Figure S1.** Phylogenetic tree of the HA gene of avian influenza A(H7N9) viruses in the 5th epidemic wave. A total of 735 H7N9 viruses collected from October 1, 2016 through September 30, 2017 were analyzed. The tree is rooted in A/Shanghai/2/2013 (H7N9). The H7N9 viruses collected from Yunnan Province are marked in red and the rest of the H7N9 viruses are marked in black.)

**Additional file 2: Table S1.** Primers, reaction system and cycling steps for real-time RT-PCR detection of Avian Influenza A(H7N9) Virus.

**Additional file 3: Table S2.** Accession number of all sequences of H7N9 virus in Wenshan City.

## Data Availability

The datasets used and analyzed during the current study are available from the corresponding author on reasonable request.

## Abbreviations

AIV: avian influenza virus
CDC: Center for Disease Control and Prevention
LPM: live poultry market
PRD: Pearl River delta
YRD: Yangtze River delta
