# Supplementary material for: Emergence of human avian influenza A(H7N9) virus infections in Wenshan City in Southwest China, 2017
Source: BMC Infect Dis. 2020 Feb 19;20:154. doi: 10.1186/s12879-020-4858-6 (PMC7031964; doi:10.1186/s12879-020-4858-6)
Supplement: Supplementary file 2 — Additional file 2: Table S1. Primers, reaction system and cycling steps for real-time RT-PCR detection of Avian Influenza A(H7N9) Virus. [file 12879_2020_4858_MOESM2_ESM.docx]

**Table S1. primers, reaction system and cycling steps for real-time RT-PCR detection of Avian Influenza A(H7N9) Virus**

RNA was extracted from specimens with the Qiagen RNeasy mini kit (Qiagen, Hilden, Germany), and Real-time RT-PCR was then performed as follow protocol.

| H7 primers and probe | | |  |
| --- | --- | --- | --- |
| Gene | Primer name | Sequences (5´- 3´ direction) | Note |
| H7 | CNIC-H7F | AGAAATGAAATGGCTCCTGTCAA | Primer |
|  | CNIC-H7R | GGTTTTTTCTTGTATTTTTATATGACTTAG | Primer |
|  | CNIC-H7P | FAM-AGATAATGCTGCATTCCCGCAGATG-BHQ1 | Probe |

The Real-time RT-PCR was performed with the AgPath one-step RT-PCR kit (Life Technologies, Carlsbad, CA, USA).

| Reaction system |  |
| --- | --- |
| Components | Volume (μl) |
| 2 × RT-PCR Master Mix | 12.5 |
| primer-forward（40μM） | 0.5 |
| primer-reverse（40μM） | 0.5 |
| Probe （20 μM） | 0.5 |
| 25xRT-pcr enzymes mix | 1 |
| Template RNA | 5.0 |
| RNase Free H2O | 5 |
| **Total** | **25** |

| The cycling steps | | | |
| --- | --- | --- | --- |
| Step | Temp. (C) | Time | Cycle |
| 1 | 45 | 10 minutes |  |
| 2 | 95 | 10 minutes |  |
| 3 | 95 | 15 seconds | 40 cycles |
| 4 | 60 | 45 seconds |  |

(1) The specimen is negative if the value of Ct was undetectable;

(2) The specimen is positive if Ct value was ≤38;

(3) The specimens with a Ct higher than 38 were repeated.
