# Supplementary material for: Emergence of human avian influenza A(H7N9) virus infections in Wenshan City in Southwest China, 2017
Source: BMC Infect Dis. 2020 Feb 19;20:154. doi: 10.1186/s12879-020-4858-6 (PMC7031964; doi:10.1186/s12879-020-4858-6)
Supplement: Supplementary file 3 — Additional file 3: Table S2. Accession number of all sequences of H7N9 virus in Wenshan City. [file 12879_2020_4858_MOESM3_ESM.docx]

| **Table S2. Accession numbers of all sequences of H7N9 viruses in Wenshan city** | | | | | | | | | |
| --- | --- | --- | --- | --- | --- | --- | --- | --- | --- |
| **Isolate name** | **Isolate ID** | **Accession number** | | | | | | | |
|  |  | **PB2** | **PB1** | **PA** | **HA** | **NP** | **NA** | **MP** | **NS** |
| A/Yunnan/32294/2017^ | EPI_ISL_285308 | EPI1102962 | EPI1102963 | EPI1102961 | EPI1102965 | EPI1102958 | EPI1102964 | EPI1102960 | EPI1102959 |
| A/Yunnan/wenshan01/2017* | EPI_ISL_339477 | EPI1360555 | EPI1360556 | EPI1360557 | EPI1360558 | EPI1360559 | EPI1360560 | EPI1360561 | EPI1360562 |
| A/Yunnan/32291/2017^ | EPI_ISL_285306 | EPI1102946 | EPI1102947 | EPI1102945 | EPI1102949 | EPI1102942 | EPI1102948 | EPI1102944 | EPI1102943 |
| A/Yunnan/32293/2017^ | EPI_ISL_285307 | EPI1102954 | EPI1102955 | EPI1102953 | EPI1102957 | EPI1102950 | EPI1102956 | EPI1102952 | EPI1102951 |
| A/chicken/Yunnan/SD193/2017^~^ | EPI_ISL_379649 | - | - | - | EPI1555563 | - | - | - | - |
|  |  |  |  |  |  |  |  |  |  |
| ^Submitted to GISAID by China CDC. | | | | | | | | | |
| *Submitted to GISAID by Yunnan CDC. | | | | | | | | | |
| ^~^Submitted to GENBANK by Harbin Veterinary Research Institute. | | | | | | | | | |
| - unavailable | | | | | | | | | |
